# Quantifying phage-bacteria dynamics *in vitro:* rapid emergence of phage-resistant mutants for *Klebsiella pneumoniae*

**DOI:** 10.17912/micropub.biology.001666

**Published:** 2025-05-30

**Authors:** Marcos Rodríguez, Irene Cantallops, Pilar Domingo-Calap, Josep Sardanyés

**Affiliations:** 1 Institute for Integrative Systems Biology (I2SysBio). C. Catedràtic Agustín Escardino Benlloch, 46980 Paterna, València, Spain; 2 Centre de Recerca Matemàtica (CRM). Edifici C. Campus de Bellaterra, Cerdanyola del Vallès 08193, Barcelona, Spain; 3 Dynamical Systems and Computational Virology (CRM-I2SysBio CSIC-Associated Unit)

## Abstract

In the quantitative description of evolving phage-bacterial systems, a central challenge lies in accurately identifying the key parameters governing the dynamics of both bacterial and phage populations. This is especially relevant in the case of multidrug-resistant pathogenic bacteria such as *Klebsiella sp* . This pathogen poses serious health problems due to antibiotic overuse, which causes the emergence of antibiotic-resistant strains and great difficulty in eradicating bacterial infections with antibiotics. Research on phage-bacteria thus becomes a very important topic to provide alternative strategies to eradicate multidrug-resistant bacteria, and thus quantitative descriptions of these processes are of paramount importance. Despite increasing research on this topic, key structural parameters of the populations, such as bacterial growth rates, the impact of phages on bacterial dynamics or the probability of emergence of phage-resistant strains, are often scarce. In this study, we investigated a battery of growth experiments for *Klebsiella pneumoniae* alone and with the presence of bacteriophage vB_Kpn_2-P4. Using mathematical models we estimate key parameters for these experiments, showing the rapid growth and emergence of phage-resistant mutants which outcompete the susceptible bacteria strains. Our results provide quantitative estimates of these processes and may be useful for understanding phage-bacterial dynamical systems and parameterizing future theoretical and computational models.

**Figure 1. Time dynamics of Klebsiella pneumoniae and the effect of bacteriophage vB_Kpn_2-P4:**
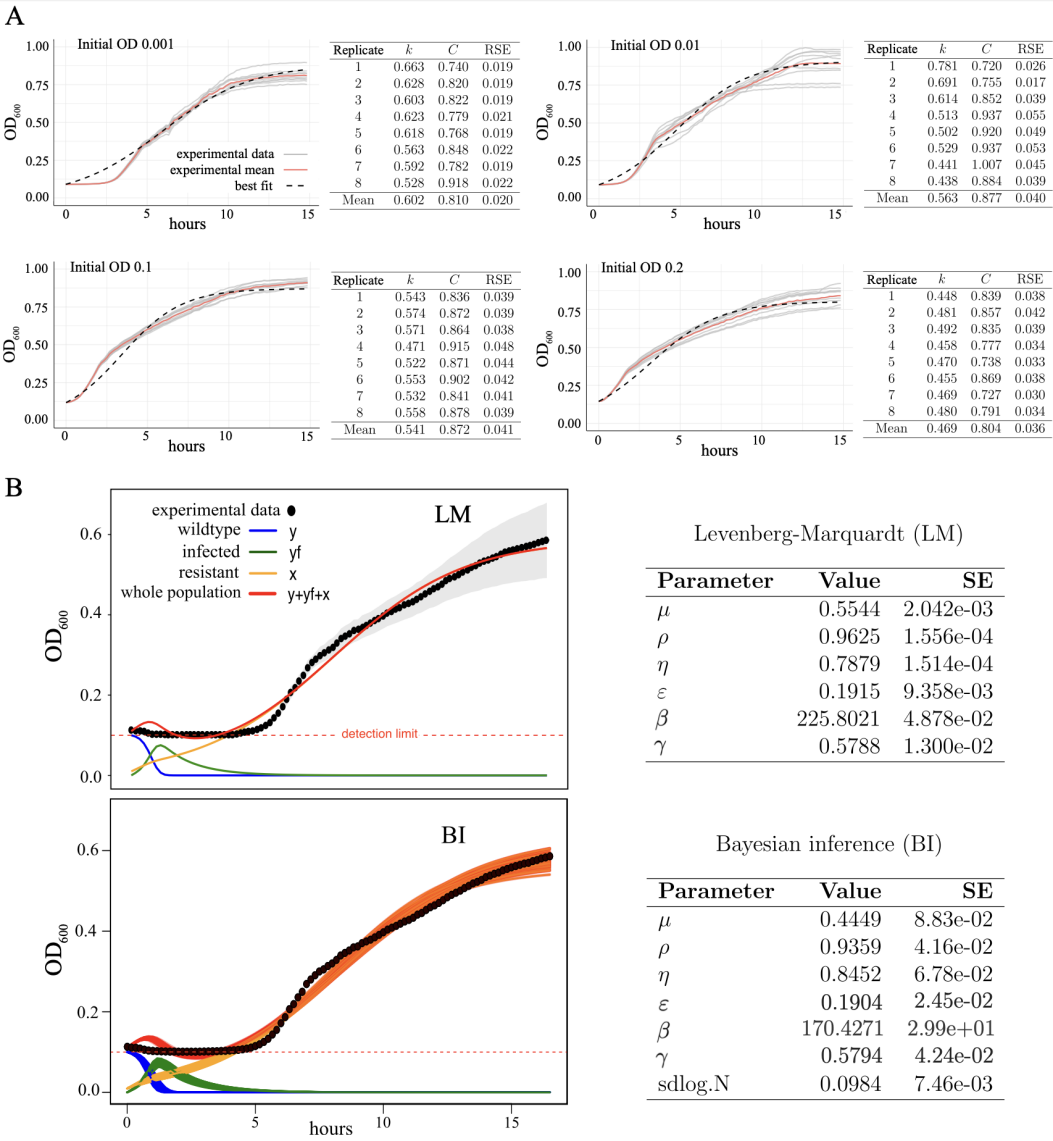
(A) Growth curves for *Klebsiella pneumoniae* ( *Kpn* ) *in vitro* using different initial populations: OD _600_ = 0.001, 0.01, 0.1, 0.2 (see Methods). Each plot displays 8 replicates (gray lines), the mean population (red lines), and the best fit (dashed black lines) obtained with Eq. (1), performed with the Levenberg-Marquardt (LM, see Methods). The tables contain the estimated values of the parameters for each initial OD (SE: Std. Error). (B) Dynamics of *Kpn* in the presence of the bacteriophage vB_Kpn_2-P4. Fits have been done with the mean populations from the experiments averaged over 21 replicas using two methods: the LM and Bayesian inference (BI). The experimental data are shown in black dots. The dynamics of the wild-type bacteria (with initial OD = 0.1) are displayed with blue lines. The dynamics of the infected and resistant bacteria are shown with green and orange lines, respectively. The sum of all the bacterial populations (red lines) has been used to compare with the experimental data. Notice that the orange lines appear overlapped with the red ones when the phage-resistant bacteria outcompete the other strains. The estimated parameter values for each case are shown in the tables at the right, using *k = 0.538* and *C = 0.887* (values obtained estimating the parameters from 15 replicates for *Kpn* dynamics alone at OD = 0.1).

## Description

Antimicrobial resistance is considered a major public health threat. For instance, the overuse and misuse of antibiotics has led to the emergence of carbapenem-resistant *Enterobacteriaceae* such as *Klebsiella pneumoniae* (hereafter *Kpn* ), becoming a critical health priority (Pintado et al., 2023). This opportunistic Gram-negative bacillus causes a wide range of infections in humans, including pneumonia, urinary tract infections, intra-abdominal infections such as pyogenic liver abscesses, wound or surgical site infections, meningitis, and bloodstream infections with the potential to precipitate sepsis (Hesse et al., 2021). Alternative strategies to combat these resistant strains are urgently needed​​, and one promising approach is phage therapy, which utilizes bacteriophages, or phages (viruses that infect bacteria), to target specific bacterial pathogens (Gan et al., 2022). Phages also possess the unique ability to co-evolve with bacterial populations, potentially overcoming bacterial resistance mechanisms, thus offering a dynamic and adaptive solution in the long term. However, the effective use of phage therapy may be limited when phage-resistant bacterial mutants evolve and proliferate during treatment (Rodriguez-Gonzalez et al., 2020). *In vitro* phage-bacteria experiments allow for a comprehensive investigation of the impact of phages on the evolutionary dynamics of bacteria and can serve to quantify such impact in terms of bacterial depletion and the selective pressures imposed by phages that can drive the evolution of phage resistance. Moreover, from a theoretical and modeling perspective, having accurate measurements of the rates of bacterial growth and the impact of phages on the population dynamics of these systems is especially important when parameterizing quantitative models brought forward to describe bacteria-phage dynamics​​. In this sense, the quantification of key biological parameters such as phage-bacteria infection rates, burst sizes, and, especially, the likelihood of emergence of phage-resistant mutants becomes relevant to investigate both mathematically and computationally more complex phage-bacteria models including these processes. As bacterial resistance to antibiotics intensifies globally, ensuring the efficacy of alternative treatments like phage therapy becomes even more crucial (Chae, 2023) and despite their long history, phage therapies are still far behind chemical antibiotic therapies. It has been previously argued that successful application of phage therapy requires a good understanding of the kinetics of phage–bacteria interactions (Cairns et al., 2009). More refined experiments and mathematical models will help to identify underlying mechanisms in more detail and to evaluate their potential (Loessner et al., 2020).

In this study, we conduct a series of *in vitro* experiments on *Kpn* treated with bacteriophage vB_Kpn_2-P4 (Ferriol-González et al., 2024). Our results contribute to a deeper understanding of phage-bacteria dynamics and its potential for combating resistant bacteria, thereby informing predictive computational models aimed at optimizing phage-based treatments. By fitting a logistic growth model to the experiments without the phage, we have quantified intrinsic growth rates and carrying capacities of the bacteria. Then, we have used these structural parameters for *Kpn* and extended the experimental results by quantifying the population dynamics of *Kpn* infected by phage vB_Kpn_2-P4 *.* For these experiments, we have used a nonlinear mathematical model and quantified key infection parameters such as phage infection rates and burst sizes. Moreover, our results show that the likelihood of the emergence or selection of phage-resistant bacteria in these experimental settings is very high.

In the first set of experiments, the concentration of *Kpn* was monitored by measuring the optical density (OD) over time for four different initial population values of *Kpn* : OD _600_ = 0.001, 0.01, 0.1, and 0.2 (for simplicity, hereafter we will use OD instead of OD _600_ ) without the presence of the bacteriophages. For each OD, we analyzed 8 replicates. The growth curves for all of the replicates and ODs were fitted independently with Eq. (2) using the Levenberg-Marquardt algorithm (see Methods). By doing so, we estimated the intrinsic growth rates ( *k* ) and the carrying capacities ( *C* ) of the bacteria expressed as OD. The estimated values for each curve are shown in Fig. 1A . Together with each replicate, the fittings were also performed for the mean growth curves. The obtained mean values and residual standard error (RSE) were: for OD = 0.001 - *k* = *0.602 h⁻¹* and *C* *= 0.810* , with *RSE = 0.020* ; for OD = 0.01 - *k* = *0.563 h ^-1^* and *C = 0.877* , with *RSE = 0.040* ; for OD = 0.1 - *k* = *0.541 h⁻¹* and *C = 0.872,* with *RSE = 0.041* ; and for OD = 0.2 - *k = 0.469 h ^-1^* and *C* = *0.804* , with *RSE = 0.036* . Variability of the growth curves across replicates increased over the length of each experiment ( Fig. 1A ). Despite this variability, our model estimated similar growth rates (and other parameters) across replicates, especially for OD = 0.01 and OD = 0.1. Similar growth rates, i.e., *0.75 h⁻¹* , have been previously obtained for phage-sensitive (antibiotic-resistant) bacteria *P. aeruginosa* grown in a murine model (Drusano et al., 2011).

In a second set of experiments, the time dynamics of *Kpn* (using an initial OD = 0.1) were monitored in the presence of the phage vB_Kpn_2-P4 *.* Figure 1B displays the fitting of the full model given by Eqs. (3.a)-(3.d), which considers the population dynamics of the susceptible and infected *Kpn* wild type (wt), together with the population of phage-resistant bacteria and the phages (see Methods). The population values obtained from this mathematical model were fitted to the mean populations of bacteria obtained from 21 experimental replicates. Since the absorbance microplate reader does not discriminate between the different bacterial strains, the experimental data was compared with the sum of all bacteria types obtained numerically from the mathematical model (red lines denoting the sum of the bacteria populations y + *y _f_* + x ). The fittings were performed using two different methods in order to seek consistent results: the Levenberg-Marquardt (LM) and Bayesian inference (BI) (see Methods). For the mathematical model, we chose an initial *OD = 0.1* for the *Kpn* wt and performed a multitude of fittings using random initial populations of resistant bacteria and phages, keeping the initial population of infected cells at zero. For the *Kpn* wild type we used structural population parameters estimated from the growth experiments with *Kpn* alone using an initial OD = 0.01. The values of *k* and *C* used for the fittings with the bacteriophages were obtained from 15 replicates using the same methodology described above, having *k* = *0.538 h ^-1^* and *C = 0.887* .

The fittings with no initial population of resistant bacteria appeared worse than those considering some small amount of resistant cells. Good fittings with the LM were obtained with an initial population of resistant bacteria of OD = 0.01 and a concentration of phages of *0.4* . For all the fittings, we used the structural parameters for *Kpn* wt estimated from the growth curves without the phages. The values of the other parameters were estimated and are shown in the tables of Fig. 1B . The parameter values obtained for the LM and the BI methods were similar, except for the burst size *β* , with *β = 225.8021 ± 0.0488***** (Std. Error) for the LM; and *β = 170.4271 ± 29.9445***** for the Bayesian inference (BI). Previous estimates of the burst size for other *Kpn* phages have been reported: *β = 31.7* for phage vB_KpnM_17-11 (Bai et al., 2023) and *β = 303* for phage BUCT631 (Han et al., 2023). Concerning the other parameters, the rate of emergence of *de novo* phage-resistant bacteria was *μ = 0.5544 ± 0.0020* (LM); and *μ = 0.4449 ± 0.0883* (BI). This can be interpreted as the probability of generating a new resistant strain from the wt duplication. As can be seen in the time series of Fig. 1B, the time dynamics display an initial decreasing value of the susceptible strain (blue line) and an increase in the infected cells (green line). The resistant strains (orange line) rapidly grow and emerge, finally becoming dominant. The infection rates obtained were *ρ = 0.9625 ± 1.556 × 10 ^-4^* (LM); and *ρ = 0.9359 ± 0.0416* (BI). The phage-induced cell death of the infected cells was very high compared to the growth rate of the susceptible *Kpn* bacteria. The estimated values for these death rates were *η = 0.7879 ± 1.514 × 10 ^-4^* (LM); and *η = 0.8542 ± 0.0678* (BI). The estimated intrinsic growth rates of the resistant strains were *γ = 0.5788* ***±*** *0.0130* (LM) and *γ = 0.5794 ± 0.0424* (BI). These results indicate that the phage-resistant strains grow at similar rates to the *Kpn* wt (cf. *k = 0.541 h ^-1^* ). This means that the resistant mutants do not pay a metabolic cost compared to the wt due to the acquisition of the resistance to the phage. Moreover, the death rate of these resistant strains is low compared to the intrinsic growth rate. The estimated values for this parameter were *ε = 0.1951* *± 9.358* × *10 ^-3^* (LM); and *ε = 0.1904* *± 0.0245* (BI).

The residual standard error for the best fit performed with the LM was *0.01766* . The error between the experimental data and the population values obtained from the mathematical model for the BI method was estimated at *0.0984 ± 0.0075* (see Table BI in Fig. 1B ).

The results presented in this manuscript are provided for a particular phage-bacteria system in *in vitro* experiments. The estimated parameters may change in different experimental settings and across bacteria strains, but they provide quantitative rates for the main processes. Such parameters may be useful in more complex *in vitro* experiments, such as those studying the impact of combining phages with antibiotics (Oechslin et al., 2017), or using cocktails with different phages against bacteria. For this latter case, the mathematical model introduced in this article could be easily extended to multiple phage populations.

## Methods

*Selection of bacterial strain*

Kpn63 is a clinical strain of *K. pneumoniae* that belongs to the capsular type KL-64 (Ferriol-González et al., 2024). It harbors an NDM-1 enzyme that confers resistance to all beta-lactam antibiotics, including carbapenems. ST-147, specifically associated with KL-64, is considered a high-risk clone circulating in Spain. Therefore, it is of interest to better understand phage-bacteria dynamics and contribute to the broader application of phage therapy in treating resistant bacterial infections, particularly in clinical settings where conventional antibiotics are increasingly ineffective.

*Bacterial growth curves*

Our experiments were performed in 96-well plates, incubated at 37°C inside a plate reader (Multiskan) for 16.5 hours to determine the bacterial growth profile based on time-lapse turbidity measurements at 10-minute intervals. Kpn63 was cultured in 3.5 mL of Luria-Bertani (LB) broth supplemented with 5 mM CaCl₂ and incubated overnight at 37°C and 180 rpm. Several experiments were performed to determine the optimal conditions of initial optical density from our bacterial suspension (OD _600_ 0.2, 0.1, 0.01, and 0.001), with 24 replicates per condition. In addition, serial dilutions from these initial bacterial suspensions were plated (150 µL spread with a Drigalski loop) and incubated overnight at 37°C in solid LB agar plates. Colony-forming units were counted to establish a correlation between OD _600_ and CFU mL-1. These experiments were performed in triplicate.

*Phage vB_Kpn_2-P4*

vB_Kpn_2-P4 is a lytic phage of *K. pneumoniae* isolated from sewage water (Ferriol-González et al., 2024). According to the crossed-infection matrix described by Ferriol-González et al., 2024, vB_Kpn_2-P4 was selected for our study due to its broadest host range in clinical strains of *K. pneumoniae* with capsular type KL-64. The starting aliquot of the phage vB_Kpn_2-P4 was stored at -80°C in SM buffer and then amplified in LB media supplemented with CaCl₂ in the same bacterial strain in which it was isolated to obtain high-titer lysates. Negative (only LB media) and positive (Kpn2 without phage) controls were used to check the efficacy of phage infection. The total volume was passed through a 0.22 µm filtration unit and concentrated using InnovaPrep equipment.

*Phage titration*

To determine the number of phage particles in our concentrated aliquots, we prepared plate cultures of vB_Kpn_2-P4 and Kpn63 in top agar overlays. We mixed 10 µL of phage serial dilutions and 100 µL of fresh Kpn63 bacterial cultures in 3.5 mL of liquid top agar at 55°C and plated after vortexing in LB agar plates. After overnight incubation at 37°C, plaque-forming units (PFUs) were counted, reaching a final titer of 1.5 × 10 ^10^ PFU mL ^-1^ . The appearance of the plaques was small and cloudy; the turbidity of the halos can result from partial bacterial lysis or the activity of phage-associated enzymes, which degrade components of the bacterial cell wall or extracellular matrix without completely lysing the bacteria.

*Time-kill curves of the lytic phage vB_Kpn_2-P4*

To study the infectivity of the phage and phage-bacteria dynamics in liquid medium, we determined the strength of lysis based on time-lapse turbidity (OD _600_ ) measurements at 10-min intervals. Our experiments were performed in 96-well plates, incubated at 37°C inside a plate reader (Multiskan) for 16.5 hours. Once we knew the correlation of CFU mL ^-1^ from our bacterial suspensions and PFU mL⁻¹from the phage aliquots, we tested different initial MOI conditions by combining 150 µL of bacterial suspension and 10 µL of phage dilution in each well. All experiments were performed with 24 replicates per condition (MOI 1, 0.1, 0.01, and 0.001).

*Modeling bacteria growth dynamics*

The growth of *Kpn* without the presence of phages was modeled using a logistic model. This model assumes an initial exponential growth of bacteria and then a population saturation due to competition for resources, i.e., nutrients. This dynamics can be modeled with the equation

dy(t)/dt&nbsp;=&nbsp;α&nbsp;y(t)&nbsp;(1&nbsp;-&nbsp;y(t)/C),&nbsp;&nbsp;&nbsp;&nbsp;&nbsp;&nbsp;&nbsp;&nbsp;&nbsp;&nbsp;&nbsp;&nbsp;&nbsp;&nbsp;&nbsp;&nbsp;&nbsp;&nbsp;&nbsp;&nbsp;(1)

where *y(t)* is the population density of *Kpn* (measured as optical density, OD) at time *t* , α being the intrinsic growth rate and *C* the carrying capacity of the system (the maximum population of bacteria the system can sustain due to, e.g., a finite amount of nutrients). This nonlinear equation can be solved analytically by integration, providing the value of the population at a given time *t* as a function of the parameters and the initial condition y(0). The solution reads:

y(t)=α&nbsp;y(0)&nbsp;exp(αt)/(α&nbsp;+&nbsp;ξ&nbsp;y(0)&nbsp;(exp(αt)-1)),&nbsp;with&nbsp;ξ&nbsp;=α/C.&nbsp;&nbsp;&nbsp;&nbsp;&nbsp;&nbsp;&nbsp;&nbsp;&nbsp;&nbsp;&nbsp;(2)

Modeling phage-bacteria dynamics

The experiments with *Kpn* in the presence of the bacteriophage vB_Kpn_2-P4 were studied with a mathematical model considering the following dynamical variables:

● *y(t)* : concentration of susceptible *Kpn* wild-type (wt) strain.

● *y _f_ (t)* : population of *Kpn* infected with phages.

● *x(t)* : population of phage-resistant *Kpn* strains.

● *ϕ(t)* : concentration of phages.

The concentrations for the bacteria are given by [CFU mL⁻¹], CFU being Colony Formation Units. The number of viral particles is given by [PFU mL⁻¹], PFU being Plaque Formation Units.

The dynamical model reads:

dy(t)/dt=k&nbsp;(1-μ)&nbsp;y(t)&nbsp;Ω(x,y)-ρ&nbsp;ϕ(t)&nbsp;y(t),&nbsp;&nbsp;&nbsp;&nbsp;&nbsp;&nbsp;&nbsp;&nbsp;&nbsp;&nbsp;&nbsp;&nbsp;&nbsp;&nbsp;&nbsp;(3.a)

$$dy_{f}\left(t\right)/dt=\rho \&nbsp;\phi \left(t\right)\&nbsp;y\left(t\right)-\eta \&nbsp;y_{f}\left(t\right),\&nbsp;\&nbsp;\&nbsp;\&nbsp;\&nbsp;\&nbsp;\&nbsp;\&nbsp;\&nbsp;\&nbsp;\&nbsp;\&nbsp;\&nbsp;\&nbsp;\&nbsp;\&nbsp;\&nbsp;\&nbsp;\&nbsp;\&nbsp;\&nbsp;\&nbsp;\&nbsp;\&nbsp;\&nbsp;\&nbsp;\&nbsp;\&nbsp;\&nbsp;\&nbsp;\&nbsp;\&nbsp;\&nbsp;\left(3.b\right)\;$$

$$dx\left(t\right)/dt=[k\&nbsp;\mu \&nbsp;y\left(t\right)\&nbsp;+\&nbsp;\gamma \&nbsp;x(t)]\&nbsp;\Omega \left(x,y\right)-\varepsilon \&nbsp;x\left(t\right),\&nbsp;\&nbsp;\&nbsp;\&nbsp;\&nbsp;\&nbsp;\&nbsp;\&nbsp;\&nbsp;\&nbsp;\&nbsp;\&nbsp;\&nbsp;\&nbsp;\&nbsp;\&nbsp;\&nbsp;\left(3.c\right)\;$$

$$d\phi \left(t\right)/dt=\beta \&nbsp;\eta \&nbsp;y_{f}\left(t\right)\&nbsp;-\&nbsp;\rho \&nbsp;\phi \left(t\right)\&nbsp;y\left(t\right),\&nbsp;\&nbsp;\&nbsp;\&nbsp;\&nbsp;\&nbsp;\&nbsp;\&nbsp;\&nbsp;\&nbsp;\&nbsp;\&nbsp;\&nbsp;\&nbsp;\&nbsp;\&nbsp;\&nbsp;\&nbsp;\&nbsp;\&nbsp;\&nbsp;\&nbsp;\&nbsp;\&nbsp;\&nbsp;\&nbsp;\&nbsp;\&nbsp;\&nbsp;\left(3.d\right)\;$$

where&nbsp;Ω(x,y)&nbsp;=&nbsp;1&nbsp;-&nbsp;(x(t)&nbsp;+&nbsp;y(t))/C,

is a logistic term introducing competition between the *Kpn* wt and the phage-resistant strains. Logistic growth involves exponential growth of bacteria at low population values and a saturation in growth as populations grow towards the carrying capacity. The parameters of the model are summarized below (units for rates are hours⁻¹ and optical density (OD) for the carrying capacity):

*k > 0* : Intrinsic growth rate of the *Kpn* wild-type strain.

μ > 0: Rate of *de novo* generation of phage-resistant bacteria from *Kpn* wt strain.

*ρ > 0* : Infection rate of wt bacteria by phages.

*η > 0* : Phage-induced death rate of infected wt bacteria.

*γ > 0* : Intrinsic growth rate of the *Kpn* phage-resistant strains.

ε *> 0* : Death rate of phage-resistant bacteria.

*β > 0* : Burst size (number of phages) from infected *Kpn* bacteria.

*C > 0* : Carrying capacity.

The model assumes that *Kpn* decay is due to the infection and lysis by the phages. Moreover, the competition between bacteria strains is only considered for those that grow, i.e., *x(t)* and *y(t),* since infected bacteria *y _f_ (t)* do not reproduce and thus do not compete for nutrients.

*Numerical tools*

The solutions of Eqs. (3.a)-(3.d) have been obtained using the 4th-order Runge-Kutta (RK) method with a constant time step size *δt = 0.01* . The model solutions were checked with a 7-8th order Runge-Kutta-Fehlberg method with automatic step size and error tolerance 10 ^-15^ , obtaining the same results. For computational purposes, we choose the 4th-order RK4. The RKF7-8 algorithm was kindly provided by the Department of Mathematics and Computer Science from Universitat de Barcelona (UB).

*Levenberg-Marquardt algorithm*

The Levenberg-Marquardt algorithm was applied for nonlinear least squares estimation of the parameters for the models used in both the growth data of *Kpn* and for the phage-bacteria data. It was implemented using the *nls.lm* function from the *minpack.lm* R package. This algorithm is highly efficient for optimizing nonlinear models by combining the Gauss-Newton method with gradient descent. To enhance robustness and convergence, we introduced random perturbations to the initial parameters at each iteration, adding or subtracting values drawn from a uniform distribution between -0.05 and 0.05. This modification allowed for broader exploration of the parameter space, improving the model fit and ensuring the selection of optimized parameters.

*Bayesian inference*

Bayesian inference (BI) provides a probabilistic framework for estimating parameters by integrating prior knowledge with experimental data, which is especially useful for models with inherent biological variability. In this study, we employed the *deBInfer* package in R to perform BI through Markov Chain Monte Carlo (MCMC) simulations. The phage-bacteria dynamics were modeled using ordinary differential equations (ODEs, see Numerical tools above), and prior distributions were assigned to each parameter based on biological insights. The inference process was enhanced using a modified Metropolis-Hastings random-walk algorithm, which rejected parameter proposals outside the prior support before solving the ODE system. This modification reduced computational complexity while maintaining precision. BI enabled us to not only estimate parameter values but also capture their uncertainty by providing posterior distributions and credibility intervals. These results were instrumental in understanding the likelihood of phage-resistant mutant emergence and other key dynamic processes, adding depth and reliability to the model.
